# Community-based nutrition education and hands-on cooking intervention increases farmers’ market use and vegetable servings

**DOI:** 10.1017/S1368980022000660

**Published:** 2022-09

**Authors:** Jessica Jarick Metcalfe, Jennifer McCaffrey, Melissa Schumacher, Caitlin Kownacki, Melissa Pflugh Prescott

**Affiliations:** 1 Department of Food Science and Human Nutrition, University of Illinois at Urbana-Champaign, 539 Bevier Hall MC-182, 905 South Goodwin Avenue, Urbana, IL 61801, USA; 2 Office of Extension and Outreach, University of Illinois at Urbana-Champaign, Urbana, IL, USA

**Keywords:** Cluster randomised trial, Mixed methods, Diet, Family, Community-based intervention, Food waste, Food resource management

## Abstract

**Objective:**

The objective of the current study was to evaluate the impact of the Market to MyPlate (M2MP) program on participants’ reported farmers’ market (FM) attitudes and shopping behaviours, frequency of serving vegetables to their families, food resource management behaviours and food security. A secondary objective was to identify facilitators and barriers to shopping at FM and food waste reduction techniques used by low-income families.

**Design:**

The current study used a mixed methods evaluation embedded within a cluster randomised trial of the M2MP intervention.

**Setting:**

The 7-week M2MP program was delivered at Extension offices and community centres in central Illinois.

**Participants:**

Participants included 120 adults and their families. Class cohorts were randomly assigned to one of three treatment groups: (1) nutrition education and cooking classes with produce allocations (PAE, *n* 39); (2) nutrition education and cooking classes only (EO, *n* 36) or (3) control group (*n* 45).

**Results:**

Compared with control participants, PAE participants were significantly more likely to report shopping at FM (*P* = 0·029) and reported serving more vegetables to their families (*P* = 0·010) (EO participants did not differ from the control group on any outcomes). There were no differences between conditions in survey-based measures of food security or food resource management behaviours. Interview results describe facilitators and barriers to shopping at FM and a variety of food waste reduction techniques (including food placement and food resource management).

**Conclusions:**

These findings suggest that fresh produce provision coupled with nutrition and culinary education can positively impact shopping and dietary behaviours.

Unhealthy dietary consumption among Americans is a major public health concern; as failure to meet dietary guidelines is associated with a wide range of chronic health issues including obesity, type 2 diabetes, metabolic syndrome, stroke and heart disease^(1–3)^. The vast majority of Americans do not meet dietary recommendations^(4)^, and individuals from families of low income are at even higher risk for unhealthy dietary intake^(5)^. Additionally, individuals from families of low income are more likely to under consume fruits and vegetables, whole grains and lean proteins and overconsume sugar-sweetened beverages, energy-dense foods and processed meats compared with individuals from middle- or high-income families^(6)^.

Recent research suggests that the promotion of farmers’ markets (FM) could have a positive influence on Americans’ dietary health, as shopping at FM is associated with increased fruit and vegetable intake^(7–10)^. FM can help address issues like limited access to fresh produce in low-income communities, which contribute to challenges individuals from families of low income face in consuming healthy diets^(8)^. One FM promotion intervention that took place in New York City found that participants reported greater consumption of fruits and vegetables, more positive attitudes towards consuming produce and higher self-efficacy for preparing and consuming produce after participating in an educational intervention at the FM^(11)^. Another study in Washington implemented FM initiatives (coupons, nutrition education and environmental interventions at FM) to improve access for food assistance programme users and found that participants who participated in FM activities reported shopping at the FM more and ate more fruits and vegetables than non-participants^(12)^. Research with food assistance program users at FM has found that high costs, limited knowledge about cooking fruits and vegetables and fresh produce spoilage concerns are all barriers to purchasing and consuming fresh produce in this audience^(13,14)^.

Though increasing Americans’ consumption of fresh produce would have positive health impacts, it is also important to acknowledge that these perishable foods are at higher risk for food waste and spoil faster than less healthy, more shelf-stable options^(15,16)^. Food waste is a major concern worldwide, as it contributes to greenhouse gas emissions and climate change^(17)^, and has a negative impact on global food security^(18)^. In developed countries like the USA, waste at the household level is the largest contributor to food waste^(19,20)^ and is also the most costly type of food waste from both an environmental and economic perspective^(21)^. Due to these concerns, experts have recently called for the implementation of interventions that target reduced household food waste^(22)^.

Currently, evidence on the relationship between income level and household food waste is mixed. Some studies at the household level have found no relationship^(23)^, or an inconsistent relationship^(24)^ between income and food waste behaviours. Other studies have found a positive relationship between waste and income, concluding that families of low income tend to waste less food than wealthier families^(25)^. Though the relationship between income and the amount of food families waste is still unclear, there is evidence to suggest that families of low income have unique attitudes about and strategies to prevent food waste. To reduce the risk of food waste (which can be even more aversive in families with constrained budgets), families of low income may be less likely to purchase unfamiliar foods^(26)^. Some researchers also suggest that the risk of food waste with highly perishable foods (like fresh produce) can deter families of low income from purchasing these items when less perishable options are available^(15,16)^.

Low food resource management skills can increase household food waste^(19,20,27,28)^, with recent research indicating that inappropriate food storage techniques (e.g. not storing foods at the proper temperature) contribute to household food waste^(29)^. Overall, food resource management techniques can improve shopping and meal planning skills (that allow consumers to maximise nutrition in a cost-effective way) and play a key role in combating food insecurity and empowering families of low income. Socio-economic status has been identified as a strong predictor of shopping behaviours, such that higher income families are more likely to purchase foods consistent with dietary recommendations^(30)^. Individuals from families of low-income report a desire to purchase more fresh produce and other healthy foods, but find this challenging given their limited grocery budgets and the higher cost associated with these items^(31)^. Food shopping practices that utilise food resource management skills (such as comparing food prices, planning meals ahead of time or using a shopping list) are associated with healthier dietary intake^(32,33)^. Experts suggest that interventions targeting improvements in food resource management skills, nutrition knowledge and meal preparation skills could be an effective way to help families of low income consume healthier diets, optimise household food budgets and avoid exacerbation of wasted food concerns^(21,31,34)^.

Given the challenges families of low income encounter in consuming healthy diets (including fresh produce), research on interventions targeting improvements in food resource management skills, nutrition knowledge and cooking and reduced food waste behaviours are warranted. Market to MyPlate (M2MP) is a community-based intervention program that teaches families of low income about nutrition, cooking, FM and food resource management. The objective of this exploratory study was to evaluate the impact of the M2MP Program on participants’ reported FM attitudes and shopping behaviours, frequency of serving vegetables to their families, food resource management, food waste behaviours and food security. A secondary objective of the current study was to better understand facilitators and barriers to shopping at FM and food waste reduction techniques used by families of low income.

## Methods

### Study design and setting

This exploratory study utilised an embedded mixed methods design, in which quantitative survey data and qualitative interview data were collected and analysed within a pilot cluster randomised trial^(35)^. The M2MP program was a 7-week family-based nutrition education and hands-on cooking intervention that randomised class cohorts (via block randomisation) to one of three experimental conditions. The block randomisation procedure randomly assigned each scheduled class time/cohort to a condition, giving each cohort equal odds of being assigned to each of the three conditions (while ensuring cohorts were spread evenly across conditions). There were a total of sixteen class cohorts, with an average of eight participants (and their families) participating in each class/time slot. The three conditions were (1) produce allocations with educational classes (PAE); (2) educational classes only (EO) or (3) control group (who did not participate in any intervention during the study, but received a delayed PAE intervention after data collection for the current study was complete). The sixteen class times/cohorts were spread evenly across different days of the week. M2MP was implemented through a partnership with the University of Illinois Office of Extension and Outreach as part of the Expanded Food and Nutrition Education Program, and classes were delivered by Extension peer nutrition educators. The M2MP classes took place between June and September 2018 in central Illinois at either a local Extension Office or at community centers with kitchen facilities. Additional details about M2MP can be found in a previous publication about the intervention^(36)^.

### Participants

Participants were recruited by Extension staff at community sites that serve low-income populations, including WIC (Supplemental Nutrition Program for Women, Infants and Children) offices and food pantries. Participants who had already taken part in an Extension nutrition education program during the past year were excluded (*n* 2), and the only inclusion criterion was that participants had to be primarily responsible for meal preparation for their families. Participants (who were not told which condition they were assigned to) self-selected class times that worked with their schedules, and researchers randomly assigned each of the sixteen class cohorts (time slots) to one of the three conditions (via block randomisation). Pre- and post-intervention survey data were collected from all consenting participants. A subsample of participants (from the two treatment conditions) who completed at least six of the seven classes participated in interviews to provide feedback about M2MP and its impact on their families. The current study was approved by the Institutional Review Board of the University of Illinois (Protocol # 17806), and all participants provided written informed consent.

### Sampling

The sample size in the current study was limited by the number of cohorts/classes, which was determined by the Extension staff’s availability to teach the M2MP classes. Since the current study was exploratory, sample size and power calculations were not conducted (because the intent of the study was to explore outcomes of interest using exploratory hypotheses, not to achieve statistical significance with a specific outcome).

### Intervention

M2MP is a 7-week nutrition education and hands-on cooking intervention targeting families of low income that used the Cooking Matters for Families curriculum^(34)^. The same curriculum and educational intervention were used in both treatment groups (PAE and EO). An assortment of fresh produce (mostly vegetables and herbs), or produce allocation, from a local farm was given to PAE participants after each class. Each weekly produce allocation was worth approximately $10 and contained approximately 5–7 produce items. EO participants were given produce coupons (of equal value to produce allocations, total $70) after the conclusion of the programme that could be redeemed at a local farm Sola Gratia Farm (that had partnered with the M2MP program) or at Sola Gratia’s stand at either of the two local FM. Both local FM accepted SNAP and WIC benefits and seasonal vouchers. During earlier sessions of M2MP, produce coupons were redeemable at the FM, while coupons given out during later sessions of the intervention (after the FM was closed for the season) were redeemable at the local farm, Sola Gratia Farm. Produce coupon redemption was tracked by M2MP researchers as coupons were used at either the FM or farm. One set of produce coupons was distributed to each family that participated. Though there were thirty-six individual adult participants in the EO condition, some individuals belonged to the same family (e.g. mother and grandmother, husband and wife), resulting in a total of twenty sets of produce coupons being distributed to participants. PAE and control participants did not receive produce coupons.

M2MP classes educated participants about local sources of produce in the community, how to use food assistance program benefits (SNAP and WIC) at FM and encouraged participants to buy, cook and eat fresh and local produce. Classes also incorporated education about a variety of food resource management techniques, including meal planning, shopping and proper food storage techniques (targeting reductions in food waste). M2MP included a nutrition education component that was based on the MyPlate dietary guidelines and also included opportunities for participants to practice hands-on cooking skills with their families. Classes were delivered by trained extension peer educators who had expertise in nutrition and cooking. Adult participants were encouraged to bring their entire family to participate in M2MP classes. When children were old enough, they participated in M2MP hands-on cooking and nutrition education activities, and childcare was provided for toddlers and babies who could not participate in classes. Recipe books (including both recipes cooked during the M2MP classes and additional recipes) were distributed at the end of the 7-week program to encourage cooking at home. Participants were required to complete at least five of the seven total M2MP classes to receive post-intervention financial incentives ($30), but all participants who were present at the last class were invited to participate in post-intervention data collection (regardless of attendance rate).

### Data collection

#### Survey data

Participants completed a pre- and post-intervention questionnaire at the beginning of the first and the end of the last M2MP class. The questionnaire included items drawn from two nutrition questionnaires (the Food and Physical Activity Questionnaire and the Food Behaviour Checklist) that had been validated in previous research^(37,38)^, as well as questions about FM that were relevant to the intervention. FM questions were developed via expert consensus among the research team members and were pilot tested with a comparable sample of adults who were eligible for food assistance. Pilot testing used cognitive interviews with probes to confirm participant understanding of survey questions.

Self-reported demographic information included participants’ gender, age, race, ethnicity, monthly food budget, number of children and nutrition assistance program participation (SNAP or WIC). The questionnaire asked participants to report on how often they served vegetables to their families, their FM attitudes and behaviours, food resource management behaviours and food security^(37,38)^. All survey items that were measured on a continuous scale had a possible range of 1–6 points, with larger scores indicating a higher frequency of the behaviour or stronger endorsement of the attitude in question. All survey outcomes were measured with a single item and included the following: frequency of serving vegetables to family, comfort level buying fresh produce at the FM, likelihood of buying produce at the FM and frequency of food resource management techniques: (1) comparing food prices to save money and (2) planning meals before grocery shopping, and frequency of food insecurity behaviours: (1) eating less to save food for family and (2) running out of money for food. One question, which asked whether participants had shopped at a FM in the last year, was measured as a binary yes/no variable. Food security questions were reverse scored so that higher scores represented greater food security. Survey data were dual-entered by two trained research assistants using a standardised electronic form. The first author then compared dual-entered data for accuracy and reconciled any discrepancies.

#### Interview data

Structured interviews were conducted with a subsample of eleven adult participants from across the two treatment conditions (PAE: *n* 6, EO: *n* 5) after the conclusion of the program. A structured protocol was used to guide interviews (see Supplemental File), which prompted participants to share their feedback about M2MP, and asked about how participating in the programme impacted their food waste and shopping behaviours. Interviews lasted between 25 and 50 min and were audiotaped (with consent) and transcribed verbatim using a professional transcription service. Transcripts and interview recordings were examined by trained research assistants in order to ensure content accuracy.

### Data analysis

#### Survey data

Descriptive statistics were calculated to characterise the sample as a whole and by condition. Differences between conditions in demographic variables were assessed using ANOVA for quantitative variables and *χ* ^2^ analyses for categorical variables. These descriptive analyses were performed using SPSS software, version 24^(39)^.

Multiple imputation methods were utilised to impute post-program survey data for those participants (*n* 40, 33 % of sample) who did not complete the post-intervention survey or were missing other data. Modern imputation methods like multiple imputation produce less biased estimates than more traditional methods such as mean substitution and list wise deletion of participants with incomplete data and can be used when a substantial proportion of data are missing^(40)^. Using multiple imputation, missing data were imputed for ten data sets using the Fully Conditional Specification method, and pooled estimates (calculated by aggregating imputed values) were used in all outcome analyses.

Multilevel modelling was used for all outcome analyses to account for the clustering of participants within the M2MP class cohorts. Multilevel linear regression analysis methods were used to assess differences between the conditions in pre- to post-intervention changes in survey scores for continuous variables. All regression analyses controlled for seasonality (month when post-program data were collected) as a class-level variable, and the following individual-level variables: gender, age, race, ethnicity, M2MP program completion, distance between home and M2MP program location, number of children, monthly food budget and food assistance program participation. Control variables were selected based on their theoretical relevance and potential to influence program outcomes, in an attempt to isolate the association between the intervention and outcomes by controlling for available demographic data. Multilevel logistic regression analyses were used to assess differences between conditions in the percent of participants who reported shopping at FM post-intervention (the only binary survey outcome). This logistic regression controlled for the same class- and individual-level variables as the linear regressions described above, and also controlled for whether participants reported shopping at FM pre-intervention. All outcome analyses used multilevel modelling and were performed using HLM software, version 8^(41)^. This was an exploratory study (with exploratory outcome analyses), and *P*-values that were less than 0·05 were considered statistically significant.

#### Interview data

Qualitative interview data were independently dual-coded by two researchers and were analysed using ATLAS.ti software (Version 8). Researchers used a hybrid deductive-inductive methodology, where the research questions informed development of the initial codebook and additional unique themes were incorporated as they emerged during the qualitative coding process^(42)^. Discrepancies in codes were discussed and were reconciled based on consensus between two coders. Coded data were assessed using a thematic analysis approach to identify common themes based on the topics and intensity of participant comments^(43)^.

## Results

**Fig. 1:**
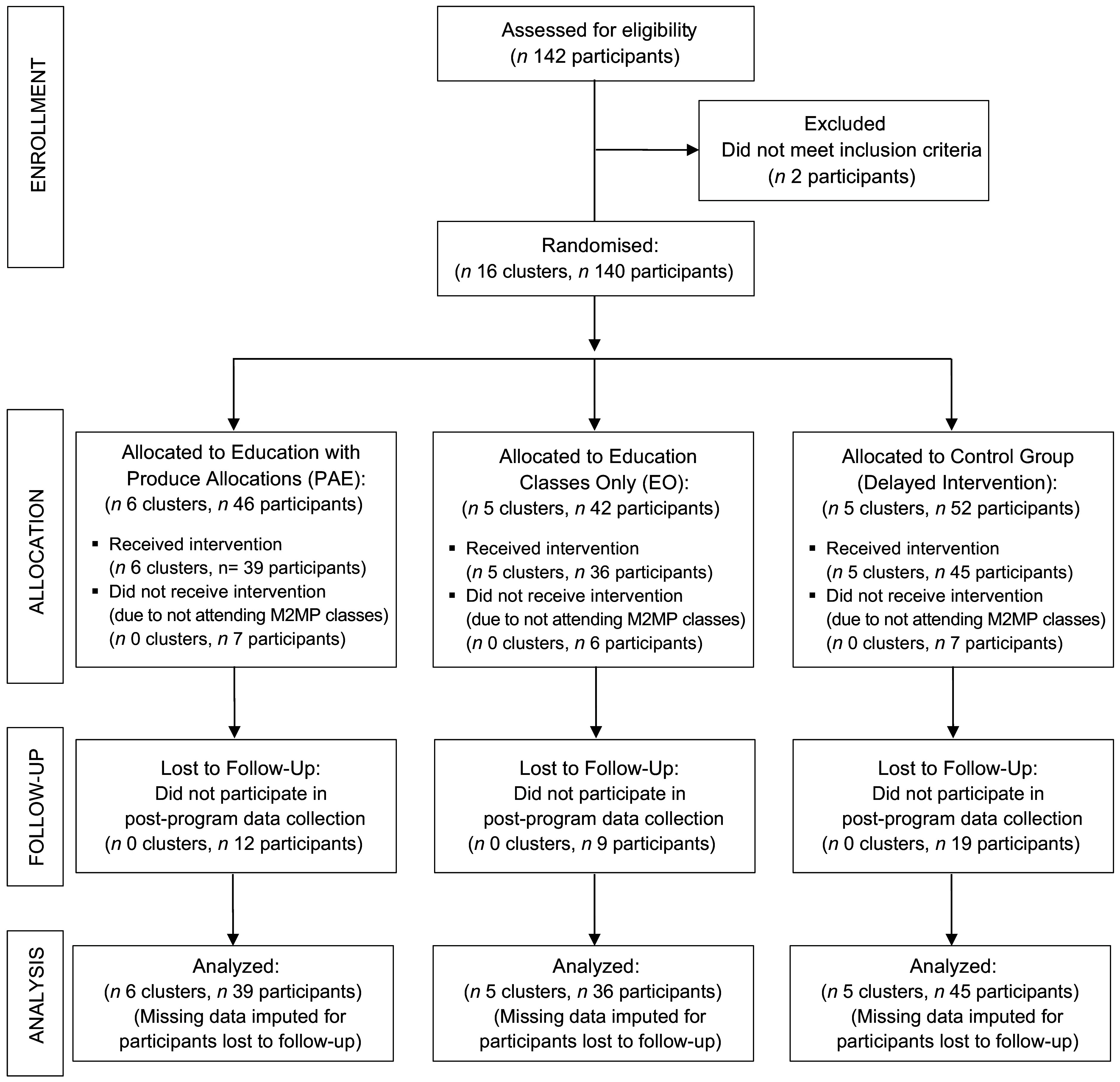
Study flow diagram in accordance with the CONSORT statement for cluster trials

A CONSORT study flow diagram depicting the number of clusters (class cohorts) and individual participants during each phase of the trial is displayed in Fig. 1. Sixteen class cohorts (140 adult participants) were randomly assigned to the PAE, EO and control conditions. A total of 120 individuals participated in the baseline survey, and eighty completed the post-program survey. Data were imputed for participants lost to follow-up, resulting in a total of 120 individual participants in the analytic sample across the PAE (*n* 39), EO (*n* 36) and control conditions (*n* 45). In addition to the survey data collected from all participants, eleven individuals (PAE: *n* 6, EO: *n* 5) participated in structured interviews to provide programme feedback.

### Sample characteristics

Descriptive statistics detailing demographic characteristics of the full sample and for each condition are displayed in Table 1. PAE participants reported larger monthly food budgets compared with participants in the control group (*P* = 0·006). PAE participants had more children (on average) than EO and control participants (*P* = 0·008). The gender distribution and participants’ average age were not significantly different across conditions. Race and ethnicity demographics for the sample were relatively diverse and did not differ significantly between conditions. There were no differences between conditions in the proportion of participants who completed M2MP data collection (took both pre- and post-surveys).

**Table 1:** Demographic characteristics of the sample as a whole and for each condition (*n* 120)

	Full sample (*n* 120)		Produce and education (*n* 39)		Education only (*n* 36)		Control group (*n* 45)		*P*
	Mean	sd	Mean	sd	Mean	sd	Mean	sd	
Age	40	12	36	10	40	11	42	14	0·076
Monthly food budget	$344	176	$407	202	$350	147	$285	156	0·006
Number of children	1·6	1·3	2·2	1·4	1·3	1·2	1·4	1·2	0·008

	Percent	*n*	Percent	*n*	Percent	*n*	Percent	*n*	
Gender									0·071
Female	72	86	82	32	75	27	60	27	
Male	28	34	18	7	25	9	40	18	
Race									0·128
Asian	14	17	13	5	25	9	7	3	
Black	34	41	26	10	33	12	42	19	
Multiracial	18	21	20	8	9	3	22	10	
White	34	41	41	16	33	12	29	1	
Hispanic/Latino	13	15	22	8	8	3	9	4	0·183
SNAP or WIC participant	47	56	59	23	33	12	47	21	0·084
Completed both M2MP Surveys	67	80	69	27	75	27	58	26	0·242

Differences in demographics between conditions were analysed using ANOVA for quantitative variables and chi-square analyses for categorical variables.

### Produce coupon redemption

Of the fourteen families that received coupons to the FM, seven (50 %) used those coupons. All (100 %) of the six families that received coupons to the farm used their coupons. Independent t-test analyses indicated that the produce coupon redemption rate was significantly higher at the farm than the FM (*P* = 0·032). Only EO participants received produce coupons; therefore, no analyses were conducted to assess differences between conditions in coupon redemption rates.

### Survey results

Multilevel linear model analyses comparing pre- to post-program changes in survey scores between conditions are presented in Table 2. Compared to participants in the control group, PAE participants reported larger increases (from pre- to post-intervention) in frequency of serving vegetables to their families (1·28 points larger increase than control, *P* = 0·010). Compared with participants in the control group, PAE participants reported larger increases (from pre- to post-intervention) in their likelihood of buying fresh produce at the FM (0·84 points larger increase than control, *P* = 0·003). There were no significant differences between the PAE and control groups in participants’ reported comfort level with buying produce at the FM, food resource management behaviours or food security. Additionally, there were no significant differences between EO participants and control group participants in any survey item responses.

**Table 2:** Multilevel linear model (MLM) analyses for differences between treatment and control groups in pre- to post-program changes in survey scores (*n* 120)

	Control group		Education only					Education & produce allocations				
	Pre-test mean	Pre-post change mean	Pre-test mean	Pre-post change mean	*β*	se	*P*	Pre-test mean	Pre-post change mean	*β*	se	*P*
Serve vegetables to family	3·52	−0·65	3·47	−0·05	0·60	0·39	0·125	3·33	+0·63	1·28	0·48	0·010
Comfortable buying fresh produce at FM	3·82	+0·15	3·89	−0·12	−0·27	0·50	0·117	3·69	+0·51	0·36	0·30	0·236
Likelihood of buying fresh produce at FM	3·07	−0·08	3·50	−0·09	−0·12	0·25	0·644	2·85	+0·76	0·84	0·27	0·003
Compare food prices to save money	3·78	+0·16	3·89	+0·10	−0·06	0·36	0·873	3·78	+0·25	0·09	0·38	0·814
Plan meals before grocery shopping	2·68	−0·17	3·02	+0·12	0·29	0·41	0·495	2·95	+0·18	0·35	0·38	0·361
Ate less to save food for family*	3·13	−0·16	3·60	+0·14	0·30	0·42	0·486	3·57	+0·21	0·37	0·40	0·362
Ran out of money for food*	3·84	+0·02	3·89	+0·01	0·05	0·39	0·896	3·74	+0·14	0·12	0·37	0·745

FM = farmers’ market.

* Reverse coded (higher scores = higher food security).

Pre-program means are displayed to provide information about average scores in each group at baseline, while betas quantify differences between experimental groups in change scores.

*P*-values displayed are for differences between the treatment groups (PAE and EO) and the control group (reference group) in pre- to post-program changes in survey scores (pre-program scores are provided for context).

All survey questions were measured on a scale from 1 to 5 points, with higher scores indicating a higher frequency/intensity of the behaviour in question.

Estimated marginal means adjusted to reflect the influence of covariates (seasonality, age, gender, race, ethnicity, number of children, monthly food budget, distance travelled to program location, M2MP program completion, and food assistance program participation) are displayed.

Multilevel logistic regression analyses comparing the proportion of participants post-intervention who reported getting fresh produce at FM in each condition are displayed in Table 3. At post-intervention, the proportion of PAE participants who reported buying produce at a FM was significantly greater (*P* = 0·029) than in the control group (controlling for pre-intervention responses and the influence of demographic covariates). There was not a significant difference between the control and EO conditions in the proportion of participants who reported getting produce at a FM post-intervention.

**Table 3:** Multilevel logistic regression analyses for differences between conditions in percent of participants who reported buying fresh produce at farmers’ market (FM) post-intervention (*n* 120)

Control group		Education only					Education and produce allocations				
Pre-program	Post-program	Pre-program	Post-program	*β*	se	*P*	Pre-program	Post-program	*β*	se	*P*
47 %	56 %	47 %	72 %	1·42	1·41	.313	49 %	85 %	8·42	3·84	0·029

*P*-values displayed are for differences between the treatment groups (PAE and EO) and the control group (reference group) in FM produce shopping post-program (accounting for covariate influence).

Percents displayed are raw frequencies which have not been adjusted for covariate influence, while betas are adjusted for covariates.

Regression analyses controlled for seasonality, age, gender, race, ethnicity, number of children, monthly food budget, distance travelled to program location, food assistance program participation, pre-program survey response for FM produce shopping and M2MP program completion.

### Interview results

#### Produce allocations

Participants reported that receiving produce allocations introduced them to new types of produce and encouraged them to purchase a greater variety of vegetables. One participant commented that, ‘when we got our [produce allocations], we would get the eggplants and stuff, and we really started to like that. So, I started buying [eggplant] now that we took the class’ (Participant 10).

All interviewed PAE participants (*n* 6) reported that they made use of the produce distributed during M2MP to cook meals at home. Two participants who received produce allocations reported that they used all the produce and did not waste any. The remaining four participants reported they had to throw away some of the produce received through produce allocations. The most common causes of produce allocation waste reported were having too much of a particular type of produce, and not knowing what to do with the produce or how to cook it. Eggplant, radishes, and turnips were the most commonly reported wasted vegetables from produce allocations. Some participants (*n* 2) also reported giving produce that they would have thrown out to other organisations (e.g. churches, food pantries) or individuals to avoid waste.

Participant feedback regarding produce allocations was generally positive, though participants did have several suggestions. The most common suggestion was to increase the quantity of more familiar and common vegetables in the produce allocations. One participant noted, ‘There were maybe a few too many sort of exotic things in [the produce allocations] … [produce allocations should] have a little more normal stuff, more universal … like onion and garlic that you can put it in pretty much anything’ (Participant 5). Participants also suggested that M2MP instructors tailor the recipes taught in class to match the produce that is sent home each week.

#### Food waste behaviours

Food spoilage was the most common reason participants had to throw away produce, though some participants also reported that they forgot to use foods before they went bad. Five participants reported positive changes in food waste behaviours after participating in M2MP, including reductions in food waste and/or using new waste reduction techniques. Six participants reported that they did not change their food waste behaviours after participating in M2MP, but three of these participants claimed that they never wasted any produce before M2MP (and therefore had no room to improve).

The most common food waste reduction techniques reported by participants are displayed with example quotes in Table 4. Participants reported using food resource management techniques, such as meal planning to use older foods first (before they spoil) and proper produce storage techniques, to reduce their food waste. Changing food placement and putting perishable items that needed to be eaten in prominent and easy to access locations was another common waste reduction technique reported by participants.

**Table 4:** Food waste reduction techniques reported in interviews (*n* 11)

Theme	Illustrative quote
Food resource management	I try to always go in the fridge and see what needs to be used up … what can we make with what we’ve got here … My wife, we’ll get something new and she’ll wanna use it and I’m like, “No, we should use the older stuff [first]” … I know, I want [to eat the new food] too, but we’ve got other stuff that we don’t wanna throw away. – PAE Participant 5, Male, Family of 2 (no children) I love that vegetable soup we made [in class] … because before the vegetables expire in the refrigerator, you can cook [that recipe] to use them up. – PAE Participant 10, Female, Family of 3 (1 child) I always thought … “I gotta put [all vegetables] in the refrigerator to keep them [from going bad].” But you don’t … We learned about not putting everything in the refrigerator [in M2MP]. – PAE Participant 8, Female, Family of 2 (1 child)
Food placement	I try to put my vegetables in the front and center of my refrigerator, so I know you gotta cook them. They’re like right there. So, I try not to waste stuff … We have a snack basket at home now, so we have more options I guess because I’ll cut up like the carrots and the celery and stuff like that or whatever and then that will be more of an obvious option. – PAE Participant 10, Female, Family of 3 (1 child) I think it was probably the fruits that, it’s like if they’re not on the counter where [my kids] can see them, if I have to stick them in the refrigerator, then they don’t seem to get eaten … And so as soon as I put em in the refrigerator, it was almost like, out of sight, out of mind type of deal … Cause like anything that we get fresh that it can stay out, so that they can access and see it. – EO Participant 1, Female, Family of 3 (2 children)

#### Shopping behaviours and farmers’ markets

Participants reported shopping for fresh produce at a variety of locations including supermarket chains, bulk stores, discount food stores, general merchandise stores (e.g. Wal-Mart), FM, food pantries and receiving free food from food assistance programs (e.g. summer meal program) or other individuals (e.g. friends’ gardens). Cost, produce quality, produce variety, convenience and proximity to the store were the most commonly reported factors that influenced where participants chose to shop. When asked whether the fruits and vegetables they purchased were locally grown, most participants (*n* 7) did not know, and the rest (*n* 4) reported that they sometimes purchased local produce.

Participants were also asked about changes in their shopping behaviours and knowledge after participating in M2MP. Some participants (*n* 4) reported that they did not change where they purchased fresh produce or how frequently they shopped after M2MP. Participants reported that M2MP taught them new strategies for shopping at the FM (e.g. asking for discounted ‘ugly’ produce) and that SNAP benefits are accepted at the FM and how to use them (*n* 4). Two participants reported shopping at the FM more and buying a greater variety of produce, while another two participants had not changed their shopping behaviours at the time of the interview, but reported that they intended to start shopping at the FM after learning about it in M2MP.

#### Facilitators and barriers to shopping at farmers’ markets

The most commonly reported facilitators to shopping at FM (Table 5) were produce quality and variety, discounts and price savings and opportunities to interact with vendors. Participants commented that produce from the FM was of noticeably better quality than supermarket produce. Participants reported that discount programmes and savings opportunities (which participants described learning about in M2MP) and interaction with vendors made them more likely to shop at the FM. Overall, participants reported that they felt welcome and comfortable at the FM.

**Table 5:** Facilitators and barriers to shopping at farmers’ markets reported in interviews (*n* 11)

Theme	Illustrative quote
*Facilitators*	
Produce quality and variety	[When we tried] the vegetables … from the farmers’ market … we liked the taste better than other [produce from grocery stores] … [the produce] was fresh, and it was tasty … [with] lots of more varieties than in just one store. – EO Participant 7, Female, Family of 5 (1 child)
Discounts and price savings	No. I still have not [gone to the farmers’ market]. But I look forward to because I [learned at M2MP] that they have a three-for-one day … that’ll make me more likely to shop [at the farmers’ market], because you really are saving on your money and maximizing your dollar. – EO Participant 2, Female, Family of 9 (8 children)
Vendor interaction	One of the benefits [of farmers’ markets] could be that you can kind of have a little bit more of an interactive experience than you might have at the store. – PAE Participant 5, Male, Family of 2 (0 children) For me if I go to the farmers’ market … it’s like I can talk to the farmer directly, whoever produced the goods … [and] we can get a close view of the products. – EO Participant 7, Female, Family of 5 (1 child) We love going to the farmers’ market. I like social events. That’s what I think of the farmers’ market as, is just a big place where people, not only are they selling foods, and produce, and things like that, but they’re socializing. It’s a nice place to meet … They have vendors there who are interacting … I mean, I like the environment. I definitely do. – EO Participant 2, Female, Family of 9 (8 children)
*Barriers*	
Timing	[The farmers’ market] is only one day a week [and has] a limited timeframe … so that’s the thing. It’s like we have to wait for it, you know. I want [to shop] somewhere we can go every [day]. – EO Participant 7, Female, Family of 5 (1 child) When I [finish work], it’s already 6:00 or 7:00 PM, so [the vendors] have already left [for the day]. – PAE Participant 8, Female, Family of 2 (1 child)
Cost and pricing	[At the] farmers’ market, I observed there is very fresh [produce], but I feel like [prices] are expensive compared to grocery stores … it’s organic, I know that, but it is not affordable. Two times [as expensive as the store] is okay, but almost five times [as much] is not. – PAE Participant 3, Male, Family of 4 (2 children) I go to the farmers’ market and I’m thinking, okay, this will be fresh vegetables in season, and I should be able to get good stuff for cheap … and then I go there, and everything’s actually double the price of [the grocery store] … I find that a lot of the farmers’ market stuff is … I mean, I understand they’re trying to kind of bring up their image a little bit to say, oh, this is some artisan-produced, locally-grown, organic. But you end up looking at it and it’s like, wow, I paid five bucks and I got this pint of beets or something … I don’t mind paying more for high quality, but … I think it’s a little extreme. It’s a little unnecessary. – EO Participant 11, Family of 3 (1 child)
Transportation and parking	I feel like a lot of people that do have the Link, and EBT, and food stamps and stuff, they go to the [grocery] store and they spend all of their money [there] cause that’s probably the only ride they had … cause nobody is gonna take the bus to go to the farmers’ market if they don’t have to … A lot of people don’t understand … but I grew up, my family, we didn’t have a car, so … we had to get what we had to get. So, I think if people had more access to [the farmers’ market] they would try it more. – PAE Participant 10, Female, Family of 3 (1 child)
Weather and seasonal closure	We would like to go to the farmers’ market that my [M2MP] teacher told me [about], but now it’s winter and it’s closed so that’s why we’re not going there. – EO Participant 7, Female, Family of 5 (1 child) Cause when it’s hot, who wants to be outside looking at vegetables? When it’s cold, who wants to be outside looking at vegetables? – PAE Participant 10, Female, Family of 3 (1 child)

Participants also commented on barriers that made shopping at the FM more difficult (Table 5). The limited hours of the FM were a deterrent for participants. Participants also reported that prices for produce at the FM (when discounts were not available) were more expensive than the grocery store. Additionally, participants reported transportation challenges and parking issues, noting that individuals who did not own a car would be less likely to shop there.

## Discussion

In this mixed methods study, participants who received a produce allocation with their educational intervention reported serving more vegetables to their families and increased their likelihood of and actual purchasing of produce at the FM relative to the control group. In interviews, PAE participants reported that produce allocations helped them incorporate what they learned in M2MP at home and introduced them to new vegetables that they had previously been unfamiliar with. In addition, we found that participants who received coupons for free produce were more likely to redeem those coupons at a local farm (100 % redemption rate) compared with the FM (50 % redemption rate). Among those who were interviewed, approximately half of participants reported improvements in food waste behaviours (either a reduction in amount of food wasted or adopting new food waste reduction techniques) after participating in M2MP. Interviews revealed that participants used a wide variety of food resource management techniques that were not fully captured by the survey. This research makes a unique contribution to the literature as the first cluster randomised trial to examine the impact of a family-based nutrition intervention with weekly produce allocations using an embedded mixed methods design.

Even though the PAE participants were more likely to report serving vegetables to their families, the majority of the interviewed participants reported having to throw away allocated produce at least once. This underscores the relevance of the food waste mitigation strategies reported by other participants, the most common being changing food placement and food resource management skills. Past research in both retail and experimental settings indicates that changing the placement of foods to make them more accessible can increase both selection and consumption of these foods^(44,45)^, but further research is needed to better understand the impact of similar behavioural economic techniques to reduce food waste in the home. Survey results suggest that pairing education with produce allocations made participants more likely to report increases in serving vegetables to their families, results which are supported by other researchers such as Smith and colleagues who found that improvements in carotenoid scores (quantifying vegetable consumption) were greatest for participants who received both education and produce to take home^(46)^. More research is needed to better understand if including familiar produce items, such as onions or garlic, would facilitate the use of unfamiliar items or serve as their competition. Regardless, findings from the current study suggest that produce allocations that include less common produce should be paired with targeted education about how to store and prepare these items.

Though food resource management (as measured by the survey) did not improve significantly, interview findings suggest that participants improved other food resource management skills (e.g. proper food storage techniques, planning meals/using recipes to reduce food waste) that have been recommended as effective techniques to reduce food waste^(21,27,28)^. Discrepancies between qualitative and quantitative findings in the current study suggest that the current EFNEP Food and Physical Activity Questionnaire may not adequately measure food resource management behaviours as they relate to food waste. Interview findings indicate that food resource management approaches used by families can include a variety of different skills and techniques, and researchers should take this into account when designing and evaluating programs. Interventions (and evaluations) targeting a variety of food resource management skills may be an effective way to conserve natural resources, while simultaneously supporting families’ ability to have healthier dietary patterns within their limited budgets.

According to our survey findings, participants’ reported FM comfort level was not significantly different between conditions, but PAE participants reported greater increases in likelihood of shopping at the FM, and more PAE participants (compared with control participants) reported actually shopping at FM post-intervention. Additionally, interview findings suggest that participants learned new strategies for shopping at FM (such as using SNAP benefits) in M2MP. Participants also reported that factors including produce quality and variety, discounts and vendor interaction made them more likely to shop at FM and challenges with transportation and parking, weather and seasonal closures, limited hours and high prices were barriers to shopping at FM. The importance of these facilitators and barriers to FM use is supported by existing research, which has found that each of these factors influences FM shopping behaviours^(8,13,14,47,48)^. Implications for improving FM use among low-income populations could include expanding hours and providing indoor options during inclement weather, offering discounts and accepting food assistance program benefits and ensuring that FM have adequate parking, are located near low-income communities and are accessible via public transportation. Studies on FM incentive programs also indicate that providing FM coupons can help reduce barriers related to the cost of shopping at FM^(13)^.

Even though interviewed participants reported feeling welcome at FM, as well as other FM facilitators, only half of the participants who received coupons for free produce at the FM redeemed them, while 100 % of those who received coupons for free produce at the farm redeemed them. This difference in redemption rate suggests that the timing and/or parking barriers may be barriers to shopping at FM for vulnerable populations. Research on FM with similar populations has also found transportation and timing/market hours to be key factors that influence FM utilisation^(48,49)^. It may be easier to arrange transportation to a local farm, which has more open hours than the weekly FM. It is also possible that farms may be a more appealing destination to either the participants or their social circle, suggesting that future interventions may benefit from incorporating farm visits. Since participants’ options of where to redeem coupons were influenced by seasonality (coupons were redeemable at the farm after the FM had closed for the season), it is not possible to discern whether the coupon redemption location (farm *v*. FM) was the sole driver of this outcome. Past research with FM coupon interventions also indicates that FM vouchers can increase feelings of autonomy and dignity among consumers of low income, while supporting social connections and fostering a sense of community^(49)^. In the future, researchers could investigate whether vouchers for local farms have similar impacts.

The current study is not without limitations. The M2MP intervention had a high attrition rate (33 %), though dropout rates were similar across conditions. It should be noted, however, that high attrition rates are not uncommon with vulnerable populations (i.e. due to challenges such as transportation that could make attending class more difficult), and the use of multiple imputation in the analysis of survey data in the current study follows best practices for interventions with high attrition^(50)^. Though the multiple imputation methods used mitigated the impact of participant attrition on survey outcomes, our interview data were limited to those who had graduated from the programme and may not adequately reflect the experiences of those who were unable to complete the programme. While we did not control for the fact that some participants came from the same family, the majority of participants from the same family did not live together (e.g. mother and grandmother), and therefore would have different responses for household related information. We did conduct sensitivity analyses for adult family members who lived together (e.g. averaging responses for a husband and wife and counting them as one participant) and found that study outcomes did not differ when we only counted each household once. Additionally, analyses of coupon redemption rates were limited by the fact that seasonal changes coincided with coupon redemption locations. Since this was an exploratory study, findings should be viewed as preliminary and should be confirmed and replicated in future research. Lastly, it should be noted that M2MP only took place in one geographic location in central Illinois, and more research is necessary to determine whether results are generalisable outside of this geographic setting.

Despite these limitations, the current study also has several notable strengths that should be highlighted. The M2MP intervention used a rigorous experimental design that implemented a cluster randomised trial with an embedded mixed methods evaluation. By including two treatment conditions (one with education only and one with education and produce allocations), researchers were able to determine that the inclusion of produce allocations was related to stronger study outcomes (in relation to serving vegetables and FM shopping). The mixed methods design allowed researchers to better understand survey-based findings when paired with qualitative findings, and interviews revealed positive outcomes (e.g. related to food waste and food resource management) that were not included in surveys. The sample for the current study was a relatively diverse low-income audience, so the generalisability of our results is not limited by participant demographics. Additionally, the current study makes a unique contribution to the literature on household food waste, as published studies that examine outcomes related to food waste are uncommon^(22)^.

## Conclusions

This embedded mixed methods study found that participants in M2MP intervention program who received produce allocations in addition to nutrition education experienced significant increases in their frequency of serving vegetables to their families and shopping at FM (relative to the control group). Families in M2MP who received coupons for free produce were more likely to redeem their coupons at a local farm compared with the FM, suggesting that incorporating local farms could be a promising avenue for future interventions. Qualitative findings suggested that the food resource management skills measured by the survey did not include the full range of techniques participants used to reduce food waste. Findings from the current study indicate that interventions and evaluations targeting a variety of food resource management skills could be an effective approach to reduce food waste, improve shopping skills and support healthier dietary intake in vulnerable populations.

## Supplementary material

For supplementary material accompanying this paper visit https://doi.org/10.1017/S1368980022000660. click here to view supplementary material
