# Supplementary material for: Community-based nutrition education and hands-on cooking intervention increases farmers’ market use and vegetable servings
Source: Public Health Nutr. 2022 Mar 21;25(9):2601–13. doi: 10.1017/S1368980022000660 (PMC9991668; doi:10.1017/S1368980022000660)
Supplement: Supplementary file 1 [file S1368980022000660sup.zip › S1368980022000660sup002.docx]

**Supplemental Interview Information – Market to MyPlate Intervention**

**Interview Details (as requested by COREQ Checklist):**

*Research Team and Reflexivity*

- The following researchers conducted the interviews:
  - [First Author, blinded for review], PhD, MPH, Postdoctoral Research Associate
  - [Last Author, blinded for review], PhD, RDN, Assistant Professor
  - [Blinded for review], MS, RD, MAPP, Coordinator of Programming and Evaluation (Extension)
    - This individual is not an author on this paper but assisted with the evaluation of M2MP
- All interviewers were female, and had extensive training and experience with program evaluation and qualitative data collection
- Interviewers established communication with participants prior to interviews (during interview recruitment)
- Interviewers explained the reasons for doing this research and interest in the research topic prior to interviews
- The following researchers coded and analyzed interview data:
  - [First Author, blinded for review], PhD, MPH, Postdoctoral Research Associate
  - [Second Author, blinded for review], MS, RD, LDN
  - [Third Author, blinded for review], MS, RDN, LDN
- These research team members all had extensive experience with nutrition and cooking education programs, and with coding and analyzing qualitative data

*Study Design*

- Thematic analysis of the content of interviews provided the theoretical approach for qualitative data collection and analysis
- All participants who met interview criteria (attended at least 6 of 7 total classes) were invited to participate in interviews (purposive sampling of participants who received high/full dose of intervention)
- Announcements were made in M2MP classes that interview opportunities were available to participants who had attended at least 6 of 7 classes, participants then signed up (volunteered) to be contacted (via phone or email) about scheduling interviews
- Researchers interviewed all participants who were eligible and volunteered, data saturation did not play a large role in recruitment efforts
- No participants dropped out or refused to participate in interviews
- Interviews took place in a private room at the Extension Office (where many participants had attended M2MP classes)
- No one besides the interviewer and interviewee were present during interviews
- Repeat interviews did not take place, and transcripts were not reviewed by participants (it was no longer possible to contact/follow up with all participants by the time transcripts had been created and checked)
- Researchers used audio recording only (not video) during interviews
- Field notes were made during or after interviews as needed
- Each interviewer practiced/pilot tested the interview protocol to ensure clarity

*Analysis and Findings*

- Two coders coded each interview independently, then met to reconcile codes
- Two coders analyzed each interview transcript. When two coders disagreed on codes and could not come to consensus, a third coder (also an author on the manuscript) would provide input to “break the tie” and help the team reach consensus
- Key themes that resulted from the thematic analysis are outlined in the manuscript (Tables 4 and 5)
- The hybrid deductive-inductive methodology is described in the manuscript, page 7
- Participants did not provide feedback on the findings (it was no longer possible to contact/follow up with all participants by the time transcripts had been coded and analyses were complete)

**Characteristics of Interview Participants (*n* = 11)**

| **Characteristic** | ***n* (%) or**  **Mean (Range)** |
| --- | --- |
| **Experimental Condition** |  |
| **PAE** | 6 (55%) |
| **EO** | 5 (45%) |
| **Gender** |  |
| **Female** | 7 (64%) |
| **Male** | 4 (36%) |
| **Household Size** | 3.7 (1 – 9) |
| **Number of Children** | 1.9 (0 – 8) |

**Market to MyPlate Interview Protocol:**

Hello, my name is _______. I’m a researcher from [blinded for review]. I’m talking with you today to find out more about your thoughts and experiences with the Market to MyPlate program. Please don’t be shy and give me your honest answers about the program. Your feedback will help us learn how the program works and how we can make it better.

1. To start out, can you tell me who else from your family participated in the Market to MyPlate program?
   1. Do all of these people live with you?
   2. How old are they? (For child family members only.)
   3. Does anyone else live with you that did not participate in Market to MyPlate?
2. Can you tell me about how participating in this program has impacted your family?
   1. Can you tell me about how participating in Market to MyPlate has impacted you?
3. What was your favorite thing about the Market to MyPlate program?
4. What was your least favorite thing about the Market to MyPlate program?
5. How do you think we should make the program better?

Now I’m going to ask you some questions about how your food related behaviors, like shopping for food and how you use food, might have changed since participating in the Market to MyPlate program. It’s okay if they haven’t changed; you can just tell me that things have stayed the same. So, let’s keep in mind that you started Market to MyPlate in [month]. To put you in that frame of mind, [insert appropriate time prompts based upon cohort month, such as kids would have just ended the school year, that was right before July 4^th^, etc.]

1. Before you started participating in Market to MyPlate in [month], where, if at all, did you typically purchase fresh fruits and vegetables?
   1. Why did you tend to purchase fruits and vegetables there?
   2. How often did you tend to purchase fruits and vegetables there?
   3. Where did other people in your family tend to purchase fresh fruits and vegetables?
   4. Do you know if the produce you purchased prior to Market to MyPlate locally grown?
   5. Had you ever used EBT/SNAP/LINK benefits to purchase produce prior to Market to MyPlate?
2. Before you started participating in Market to MyPlate, how often, if at all, were you cooking meals at home for your family?
   1. How often did other family members help prepare meals at home?
3. Before you started participating in Market to MyPlate, what kinds of vegetables, if any, were you eating?
   1. What about green vegetables?
   2. Were these the types of vegetables that you served to your children?
   3. Were these vegetables fresh, canned or frozen?
   4. Were these vegetables grown locally?
4. Before you started participating in Market to MyPlate, how often were you eating vegetables?
   1. How often do you think your children were eating vegetables before Market to MyPlate?
5. Before you started participating in Market to MyPlate, how often do you think you ended up throwing vegetables away in the garbage?
   1. Why did you end up having to throw them away?
   2. What kinds of vegetables did you most often end up throwing in the garbage [i.e. greens, orange, starchy, etc.]?
      1. Were they most often fresh, frozen or canned?

The rest of the questions that I will ask you are about your experiences at the current time and date, now that you’ve completed the Market to MyPlate program.

1. Now that you’ve completed the program, where, if at all, do you typically purchase fresh fruits and vegetables?
2. Why do you tend to purchase fruits and vegetables there?
3. How often do you tend to purchase fruits and vegetables there?
4. Where do other people in your family tend to purchase fresh fruits and vegetables?
5. Is the produce you purchase now after Market to MyPlate locally grown?
6. Do you use EBT/SNAP/LINK benefits to purchase produce now after Market to MyPlate?
7. Now, after participating in Market to MyPlate, how often, if at all, are you cooking meals at home for your family?
   1. How often do other family members help prepare meals at home?
8. Are there things that make it easier for you to cook at home?
   1. Can you talk about these things?
9. Are there things that make it difficult for you to cook at home?
   1. Can you talk about these things?
10. Now, after participating in Market to MyPlate, what kinds of vegetables, if any, are you eating?
    1. What about green vegetables?
    2. Are these the types of vegetables that you serve to your children?
    3. Are these vegetables fresh, canned or frozen?
    4. Do you know if these vegetables are grown locally?
11. Now, after participating in Market to MyPlate, how often are you eating vegetables?
    1. How often do you think your children are eating vegetables?
12. Now, after participating in Market to MyPlate, how often do you think you end up throwing vegetables away in the garbage?
    1. Why do you end up having to throw them away?
    2. What kinds of vegetables do you most often end up throwing in the garbage [i.e. greens, orange, starchy, etc.]?
       1. Are they most often fresh, frozen or canned?
13. Have you prepared some of the recipes that you learned in Market to MyPlate?
    1. How often do you prepare recipes that you learned in Market to MyPlate
    2. Have you prepared any new receipes since participating in Market to MyPlate?
    3. Where do you get other recipes from?

**The next questions are about shopping at farmers markets.**

1. Are there things that make it easy to shop at farmers markets?
2. Are there things that make it difficult to shop at farmers markets?
   1. [Probes will vary based on whether or not family shops at farmers markets from answers in Questions 10-11.]
      1. Do I understand you right, you feel it is easy to shop at farmers markets, but you just prefer not to shop there? Why?
      2. What kinds of things make it difficult to shop at farmers markets?
      3. How welcome do you feel at farmers markets?
3. What kinds of things would make you more likely to shop at farmers markets?
4. What sorts of foods or things do you buy at farmers markets?

**FOR EDUCATION ONLY PARTICIPANTS:**

1. I’d like you to think back to the coupons for Sola Gratia that you received at the end of M2MP.
   1. Have you used the coupons?
   2. Why not?
   3. What sorts of Produce did you buy with the coupons?
   4. Did you prepare any of the produce?
   5. How did you prepare the produce?

**FOR PRODUCE RECEIVING PARTICIPANTS**

1. I’d like you to think back to the free produce that you received during Market to MyPlate. What did you do with most of that produce?
   1. How often did you cook the produce?
      1. What kinds of things did you do make with the produce?
   2. Did the recipes from Market to MyPlate help you make use of the produce?
      1. If so, which ones? Why?
      2. If not, why?
   3. How often did you end up throwing the produce away?
      1. Why do you think you ended up having to throw the produce away?
2. Your answers have been really helpful today! What do you think is the most important thing that I should remember from talking with you today?

Thank you so much for your time and feedback!
